# Papain Ameliorates the MPAs Formation-Mediated Activation of Monocytes by Inhibiting Cox-2 Expression via Regulating the MAPKs and PI3K/Akt Signal Pathway

**DOI:** 10.1155/2018/3632084

**Published:** 2018-10-16

**Authors:** Xianming Fei, Wufeng Yuan, Yan Zhao, Huan Wang, Shi Bai, Qinghua Huang

**Affiliations:** ^1^Center of Laboratory Medicine, Zhejiang Provincial People's Hospital and People's Hospital of Hangzhou Medical College, Hangzhou 310014, China; ^2^Department of Cardiology, Zhejiang Provincial People's Hospital and People's Hospital of Hangzhou Medical College, Hangzhou 310014, China; ^3^School of Medicine, Taizhou University, Taizhou 317000, China; ^4^Department of Endocrinology, Zhejiang Provincial People's Hospital and People's Hospital of Hangzhou Medical College, Hangzhou 310014, China

## Abstract

Monocytes activation and subsequent inflammatory response mediated by monocyte-platelet aggregates (MPAs) formation play the key roles in the early pathogenesis of atherosclerosis (AS). Exploration of novel drugs to ameliorate MPAs formation-mediated monocytes activation would be helpful for the treatment of AS patients. Papain has definite pharmacological effects including antiplatelet, thrombolysis, and anti-inflammation. However, its effect on MPAs formation and the following monocytes activation remains vague. This study aimed to illustrate the underlying mechanisms of papain on MPAs formation-initiated monocytes activation* in vitro*. In this study, Papain, Cox-2 inhibitor (NS-398), and NF-*κ*B agonist (TNF-*α*) were used as the treating agents, respectively. MPAs formation and activated monocytes were measured by flow cytometry (FCM). Cox-2 mRNA, MCP-1, and proteins of Cox-2 and NF-*κ*B signal pathway were detected by qRT-PCR, ELISA, and western blotting, respectively. As we observed, papain exhibited the powerful inhibitory effects on thrombin-mediated MPAs formation and monocytes activation in a concentration-dependent manner as what Cox-2 inhibitor demonstrated. However, the inhibitory tendency was significantly reversed by TNF-*α*. We also discovered that both Cox-2 mRNA and protein expression as well as the release of MCP-1 of monocyte was inhibited by either papain or NS-398, but TNF-*α* stimulated Cox-2 expression and release of MCP-1. The results of western blotting assay indicated that thrombin-mediated proteins expression of MAPKs and PI3K/Akt signal pathway was inhibited by papain and NS-398. However, TNF-*α* notably abated the inhibitory effects of papain on the process of MPAs-initiated monocytes activation. Our findings suggest that papain can inhibit the MPAs formation-mediated activation of monocytes by inhibiting the MAPKs and PI3K/Akt signal pathway.

## 1. Introduction

Atherosclerosis (AS) is the major pathologic base of coronary artery disease. Patients with AS will show no symptoms at the beginning of AS development, but it can gradually progress into coronary artery disease that is regarded as the leading cause of deaths worldwide [1]. The atherosclerotic process is highly associated with inflammatory response in the endothelial cells of the vessel wall which results in the generation of atheromatous plaques in the arterial tunica intima [2]. Study has demonstrated that the formation of monocyte-platelet aggregates (MPAs) by binding of activated platelet to monocytes is the major risk factor of cardiovascular events, which would induce monocytes activation and the release of proinflammatory cytokines/molecules, and subsequently facilitates the formation of AS [3]. Formation of MPAs is an established link between inflammation and thrombosis in acute coronary syndromes and related disorders [4]. Therefore, investigation of the pathogenesis of monocytes activation induced by MPAs formation and looking for the inhibitor of activated monocytes would be of great practical significance.

It has been confirmed that platelets are the initiator and mediator of inflammatory response of vessel wall [5]. Activated platelets initiate the expression of P-selectin which has been verified in the condition of acute coronary syndromes [6]. The binding of P-selectin to P-selectin glycoprotein ligand type 1 (PSGL-1) contributes to the interaction between platelets and monocytes which induces monocytes activation and the expression of cytokines, chemokines, and adhesion molecules, resulting in the promotion of atherosclerotic lesion formation [5, 7]. MPAs accumulation has been observed in the progression of cardiovascular disease and regarded as a major biomarker for cardiovascular risk [8]. Previous study indicates that MPAs are significantly elevated in acute and unstable atherothrombosis [9], implying that measurements of MPAs might be used as the evaluating indicator in early stage of AS disease. MPA formation alters the phenotype of monocytes along with the increase of Mac-1 expression and activation, and the enhancement of Mac-1 can bind to platelet fibrinogen receptor (glycoprotein IIb/IIIa receptor) and maintain the adhesion of platelets and monocytes and monocytes activation in turn [10]. Under proinflammatory conditions, the enhancement of MPAs subsequently expands the pool of circulating monocytes in a cyclooxygenase-2 (Cox-2) dependent manner [11]. MPAs always serve as the sensitive marker of platelet activation, and activated platelet-mediated monocytes activation promotes monocyte-evoked inflammatory response by regulating nuclear factor kappa B (NF-*κ*B) pathway and the release of several inflammatory cytokines such as monocyte chemoattractant protein 1 (MCP-1) by TLR4 signal pathway in cardiovascular disease [12, 13]. Dixon DA et al. reported that MPAs binding of P-selectin to PSGL-1 can trigger outside-in signaling that leads to NF-*κ*B activation and Cox-2 transcription [4]. And Cox-2 regulates PGE2 production induced by TNF-*α*, promoting the development and progression of chronic inflammation response [14]. Thus, the binding of activated platelet to monocyte and subsequent monocytes activation would have essential effect on the development of atherothrombosis by integrating multiple inflammatory signaling pathway, and interruption of signaling pathways such as NF-*κ*B-Cox-2 pathway that are triggered when platelets adhere to and activate monocyte may be a new target for molecular intervention [4]. Therefore, Cox-2 might play an important role in the MPAs-medicated inflammation response resulting from monocytes activation, and it would be able to prevent the process of inflammation by inhibiting the upstream NF-*κ*B signal pathway of Cox-2 expression initiated by MPAs formation.

Several types of protease preparations have been used to improve platelet aggregation and thrombosis-related diseases. Orally used bromelain, a thiol protease, can lead to the decreases of human platelet aggregation, adhesion of platelet to endothelial cells, and thrombus formation in rat vessels [15]. Cysteine protease cathepsins is required for extracellular matrix (ECM) remodeling and implicated in the development and progression of atherosclerotic cardiovascular diseases [16]. It is confirmed that inflammatory cells and activated vascular cells such as monocytes/macrophages can induce the expression of cathepsins or other proteases to degrade extracellular matrix in the arterial wall, resulting in atherogenesis [17]. Papain, as a member of cysteine protease enzyme family, is always used to remove dead or contaminated tissue in acute and chronic lesions [18, 19]. On the basis of mass concentrations, both papain and trypsin were as effective as bromelain in preventing platelet aggregation and reducing the platelet adhesion to the endothelial cells [16]. Papain-like cysteine peptidases participate in diverse of physiological processes including antigen presentation, hormone processing, and cardiovascular events [20]. The level of cystatin C, a papain-like cysteine proteases, presents pathophysiological associations with poor cardiovascular disease outcome [21]. Papain-hydrolyzed peptides isolated from pork meat are advantageous to prevent atherothrombosis [22]. Our previous studies also indicated that papain has some inhibitory effect on platelet activation [23] and MPAs formation [24]. These studies demonstrate that papain seems to be an intervening agent in the pathogenesis of platelet activation or MPAs-mediated inflammation response. However, whether papain can regulate inflammation response and the relative mechanisms related to NF-*κ*B signal pathway of activated monocytes initiated by MPAs formation remains unclear. In this study, we aimed to explore the effects of papain on monocytes activation mediated by MPAs formation and further to elucidate the primary mechanisms of the action.

## 2. Materials and Methods

### 2.1. Participants

Thirty healthy volunteers, including 17 males and 13 females aged from 20 to 27 years old, were recruited for this study, and all subjects had taken no medicine in two weeks before sample collection. For experiment requirements, 20 to 25 milliliters venous blood from each volunteer was collected in the morning after starving for 8 hours or more according to the routine procedures. This study was approved by local ethics committee on the use of human samples for research of the hospital, and the written informed consent of all volunteers has been obtained.

### 2.2. Platelet Isolation

Whole blood was centrifuged at 120 x* g* for 10 mins and the platelet rich plasma (PRP) was collected. Then the PRP was gently mixed with 0.5 *μ*mol/L prostaglandin E1 and 6250UPL sodium heparin. Subsequently, the mixture was placed for 10 mins at 30°C and centrifuged at 500×*g* for 10 mins. The pellet was resuspended and washed with platelet washing buffer for once and centrifuged at 500×*g* for 10 mins again. Next, the pellet was resuspended again with Tyrode's buffer, and the platelet was counted by an automatic hematology analyzer (Mindray, BC-6900). Eventually, the platelet was prepared as a solution with the platelet count of 2×10^11^ /L for the following experiments.

### 2.3. Cell Culture and Treatment

Human THP-1 cells were purchased from American Type Culture Collection (ATCC) and cultured in RPMI 1640 medium (Life Technologies, 23400021) containing 10% FBS (Life Technologies, 10099) and incubated at 37°C in a humidified atmosphere containing 5% CO2 (Thermo, 3111). Total of 75 *μ*L THP-1 cell suspension (5×10^7^ cells/L) was added to 75 *μ*L platelet suspension and mixed gently. In control group, the mixture of THP-1 and platelet was treated with saline or isotype antibodies. In platelet activation group, the mixture was administrated only with 100 U/L of thrombin (final concentration, Siemens Healthcare Diagnostics Products GmbH). In papain group, the mixture with 100 U/L of thrombin was treated with 50, 100, and 200U/L papain, respectively. In Cox-2 inhibitor group, NS-398 was added to the mixture of THP-1, thrombin, and platelet. To observe the effect of NF-*κ*B agonist, TNF-*α* was added to the cocultured system in the presence of 100 U/L of thrombin and 200U/L of papain. The mixture in each group was incubated at 37°C for 30 mins and prepared for the following detection.

### 2.4. Flow Cytometry Detection

Human THP-1 cells were mixed gently with equivalent platelet solution and added to 50, 100, and 200U/L of papain at 37°C for 10 mins, respectively. For the measurements of monocyte-platelet aggregates (MPAs), the mixture in each group was incubated with CD14-PE antibodies (rabbit anti-human monoclonal antibody, Bioss), CD41-APC antibodies (mouse anti-human monoclonal antibody, eBioscience), and isotype matched controls for 20 mins at room temperature. After performing the detection with a flow cytometry (FCM, BD, C6), MPAs events were gated by identifying both CD14 and CD41 positive cells. In the process of activated monocyte detection, samples were labeled by CD11b-FITC antibody (mouse anti-human monoclonal antibody, eBioscience). Viable CD11b^+^ monocytes were analyzed and counted by the FCM.

### 2.5. ELISA Detection

In each coincubated group, supernatant was collected, and the release level of MCP-1 from THP-1 cells was measured by ELISA (NeoBioscience, EHC113) according to the manufacturer's instruction. Briefly, 100*μ*l of samples and standard were added to the ELISA plate and incubated at 37°C for 90 mins. After washing with cleaning solution for 5 times, biotinylated MCP-1 antibody was added and incubated for another 60 mins at 37°C. Subsequently, enzyme conjugate was added to the mixture, standing for 30 mins at 37°C after washing for 5 times. Finally, 100*μ*l of chromogenic substrate TMB was added and incubated for 15 mins at 37°C in darkness. After terminating reaction, the OD value of all samples was measured at the wavelength of 450 nm by microplate reader (Sigma, 1-15K). Eventually, the level of MCP-1 was calculated based on the equation of standard curve.

### 2.6. Real-Time Polymerase Chain Reaction Detection

Total RNA from the samples were isolated in the light of the protocol of Trizol reagent (Roche). One microgram (1 *μ*g) total RNA was reversely transcribed into cDNA using SuperRT cDNA Synthesis Kit cDNA (CW Biotech, CW0741S, China). The level of Cox-2 gene was evaluated by performing real-time PCR utilizing UltraSYBR Mixture (High ROX) (CW Biotech, CW2602M, China). Primer sequences of Cox-2 are as follows: forward, AACGATCCCTCCCTTACCAT; reverse, GTTTAGACGTCCGGGAATTG. *β*-actin (forward, GATGAGATTGGCATGGCTTT; reverse, GTCACCTTCACCGTTCCAGT) served as the internal control. Relative expression of Cox-2 gene was calculated according to 2^−ΔΔCt^ method.

### 2.7. Western Blotting Analysis

In brief, all samples were centrifuged and the pellet was resuspended using RIPA cell lysis solution containing protease inhibitors (Beijing Dingguo Changsheng Inc). After being shaken vigorously for 30 mins at 4°C, the lysis solution was centrifuged at 12, 000 x* g* for 10 mins at 4°C. The supernatant was collected, and quantitative protein analysis was performed by BCA kit according to the manufacturer's instruction. Total 30 *μ*g of protein was used to perform SDS-PAGE assay. Different proteins were detected by specific primary antibodies. Antibodies against Akt, p-Akt, JNK, p-JNK, p38, and p-p-38 (rabbit anti-human monoclonal antibody) were purchased from Cell Signaling Technology. *β*-actin, ERK, and p-ERK antibodies (rabbit anti-human monoclonal antibody) were from Santa Cruz Biotechnology. Cox-2 antibody (rabbit anti-human monoclonal antibody) was obtained from Abcam. Relative protein analysis was processed based on Quantity One software.

### 2.8. Statistical Analysis

Each experiment was independently performed at least three times. Data were first tested for normality by Kolmogorov-Smirnov test (K-S test), and normally distributed data were presented as the mean ± SD. Relative protein level was measured using Quantity One software. The difference of calculated results was compared with one-way ANOVA test or t-test. All statistical analyses were performed with SPSS software (version 17.0). P value of less than 0.05 was considered as statistically significant.

## 3. Results

### 3.1. Papain Inhibits Thrombin-Initiated MPAs Formation and Monocytes Activation

**Figure 1:**
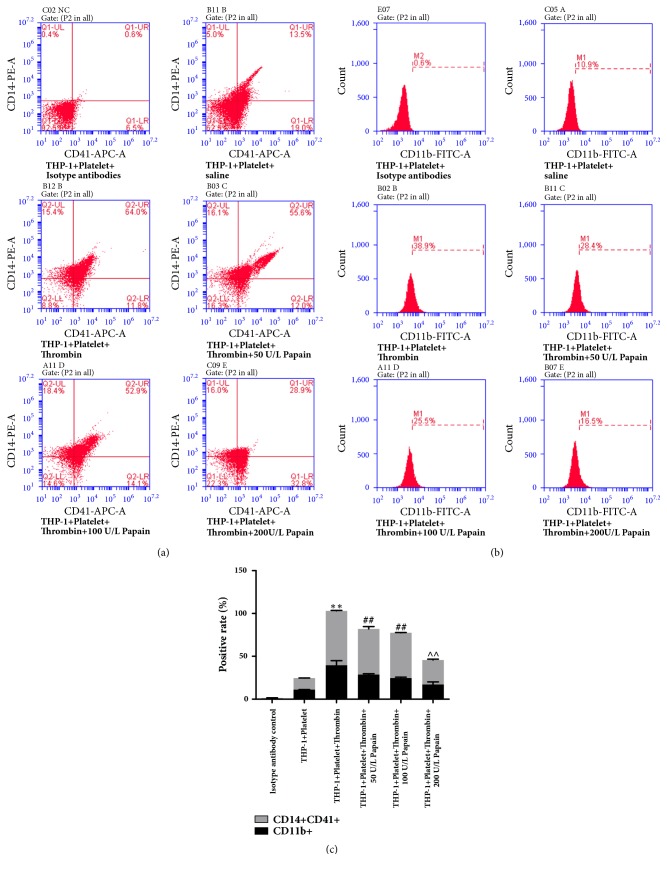
**Effect of papain on the formation of monocyte-platelet aggregates and monocytes activation. **(a) Gating strategy for the quantification of MPA subsets in the coculture system of THP-1 cells and activated platelets by flow cytometry (FCM). MPAs was evaluated by expression of CD14 and CD41. CD14^+^ cells suggested being monocytes, and activated platelet was identified by CD41 expression. (b) Surface marker of monocyte activation was measured by FCM detection. Activated monocytes were stained with CD11b. (c) Results of quantitative analysis of CD14 and CD41 as well as CD11b positive cells in different concentrations of papain groups. K-S test showed that data were normal distribution in all groups (P>0.05). *∗* indicates thrombin-treated group versus control group. ^#^ indicates papain and thrombin-treated group versus single thrombin-treated group. *∗∗*p<0.01, ^##^p<0.01.

To determine the effects of papain on MPAs formation, we cocultured THP-1 cells and activated platelets induced by thrombin in different concentrations of papain (50, 100, and 200 U/L). After performing flow cytometry (FCM) with CD14-PE and CD41-APC monoclonal antibodies, we found that the percent of CD14^+^CD41^+^ cells significantly increased (63.5±0.5% versus 13.5±0.2%, P<0.01), indicating that MPAs formation was vividly facilitated. Under the treatment with 50 and 100U/L of papain, the levels of MPAs formation were moderately suppressed (53.2±2.8% and 52.9± 0.4% versus 63.5±0.5%, P<0.01 respectively), but 200U/L of papain significantly inhibited MPAs formation (28.7±0.9% versus 63.5±0.5%) (Figures 1(a)–1(c)). Activated platelets could promote the formation of MPAs which initiated the activation of monocytes. As observed in the results of FCM detection by CD11b-FITC antibody, CD11b showed a 3.5-fold increase approximately in the cocultured system of THP-1 cells and platelets under the treatment with thrombin (Figures 1(b) and 1(c), 39.6±5.3% versus 11.0±0.2%, P<0.01, the third column versus the second one). However, when the cocultured system was treated with papain, about 30% of CD11b-positive cells (activated monocytes) were inhibited at the concentration of 50 and 100U/L compared with those in single thrombin-treated group (28.7±2.0% and 24.5±1.2% versus 39.6±5.3%, P<0.01 respectively), and about 57% of activated monocytes were suppressed under treatment with 200U/L of papain (Figures 1(b) and 1(c), the sixth column versus the third one). These data demonstrate that papain administration notably ameliorates the formation of MPAs and the subsequent elevation of activated monocytes.

### 3.2. MPAs-Mediated Release of Inflammation Cytokines from Activated Monocytes Is Inhibited in the Presence of Papain

**Figure 2:**
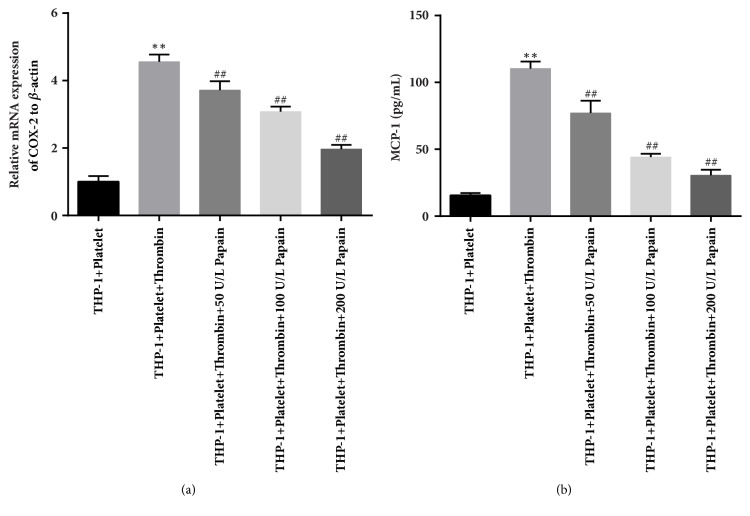
**The expression of inflammatory molecules of monocytes in the presence of papain. **(a) Transcriptional level of Cox-2 in control, thrombin and papain-treated groups were detected by performing qRT-PCR assay. (b) The expression of MCP-1 in different group was tested by using ELISA kit. K-S test showed that data were in normal distribution in all groups (P>0.05). *∗* indicates thrombin-treated group versus control group. ^#^ indicates papain and thrombin-treated group versus thrombin-only-treated group. *∗∗*p<0.01, ^##^p<0.01.

Previous study has indicated that monocyte-platelet attachment could cause the increase of surface marker and the release of inflammatory factors of activated monocytes [11]. Thus, to further confirm the inhibitory effect of papain on MPAs formation-induced inflammatory response in monocytes, we evaluated the expression of the downstream proinflammatory and adhesive molecules potentially related to AS. Given that the levels of Cox-2 and MCP-1 are the indicators of activated monocytes in many diseases such as cardiovascular disease, we determined the expression levels of Cox-2 and MCP-1 in the presence of both thrombin and papain with qRT-PCR and ELISA, respectively. The results of qRT-PCR detection showed that the transcriptional level of Cox-2 was markedly increased in the presence of thrombin, whereas the expression of Cox-2 mRNA was inhibited by papain in a concentration-dependent manner (Figure 2(a)). Similarly, thrombin-induced expression of monocyte MCP-1 in the supernatant was effectively suppressed at different concentrations of papain (Figure 2(b)). From the results above, we conclude that papain really has remarkable inhibitory effects on the formation of MPAs, MPAs formation-induced activation of monocytes, and the secretion of inflammatory molecules from activated monocytes.

### 3.3. TNF-*α* Reverses the Protective Effects of Papain on MPAs Formation-Evoked Monocytes Activation

**Figure 3:**
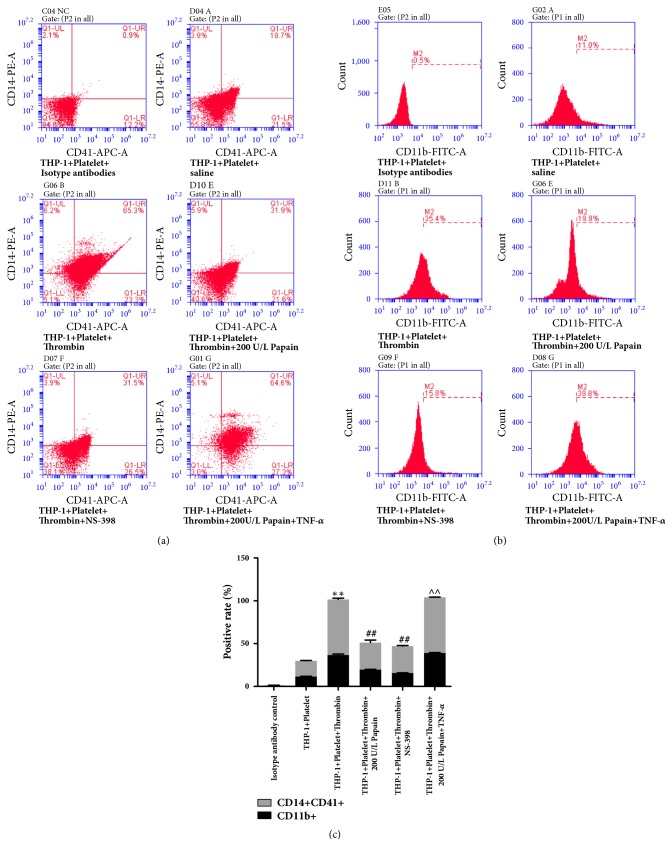
**Impact of TNF-** **α** **on the protective effect of papain on MPAs formation and monocytes activation. **(a) Gating strategy for the quantification of MPA subsets in the coculture system of THP-1 cells and activated platelets by flow cytometry (FCM). MPAs were evaluated by expression of CD14 and CD41. CD14^+^ cells suggested being monocytes, and activated platelet was identified by CD41 expression. (b) Surface marker of monocyte activation was measured by FCM detection. Activated monocytes were stained with CD11b. (c) Results of quantitative analysis of CD14 and CD41 as well as CD11b positive cells in control and thrombin, papain, NS-398 and TNF-*α*-treated groups. K-S test showed that data were in normal distribution in all groups (P>0.05). *∗* indicates thrombin-treated group versus control group. ^#^ indicates papain and thrombin-treated or NS-398-treated groups versus single thrombin-only-treated group. ∧ indicates papain+TNF-*α*-treated group versus papain or NS-398-treated groups. *∗∗*p<0.01, ^##^p<0.01, ∧∧p<0.01.

The increase of Cox-2 level is an indicator for activated platelets-induced formation of MPAs and monocytes activation [11]. In this study, we discovered that thrombin-mediated upregulation of monocyte-platelet attachment (CD14^+^CD41^+^ cells) was effectively inhibited by 200U/L of papain and Cox-2 inhibitor NS-398 (31.6±0.3% and 31.7±0.5% versus 65.0±1.0%, P<0.01, respectively) in the cocultured system of THP-1 cells and activated platelets (Figures 3(a)–3(c), the fifth and fourth columns versus the third one, respectively), which indicated that Cox-2 and papain were potent inhibitors for MPAs formation. Based on the powerful blocking effect of papain on Cox-2 expression in the above results, we speculated that the papain would improve the progression of MPAs formation primarily through inhibiting the expression of Cox-2. Evidence indicates that activated platelets induce the expression of Cox-2 and MCP-1 in monocytes mainly via activating NF-*κ*B signaling [13]. Thus, we explored whether papain could regulate the formation of MPAs by NF-*κ*B signaling pathway. Upon stimulation with TNF-*α* (NF-*κ*B agonist), papain-mediated downregulation of CD14^+^CD41^+^ cells was drastically neutralized and close to the level of thrombin-stimulated group (65.0±0.2% versus 65.0±1.0%, P<0.01) (Figures 3(a)–3(c)). Next, we further evaluated the role of Cox-2 and NF-*κ*B signal pathway in the course of monocytes activation. The results indicated that both papain and Cox-2 inhibitor (NS-398) significantly inhibited the levels of thrombin-induced CD11b expression (19.6±0.3% and 15.6±0.3% versus 36.5±1.3%, P<0.01 respectively). However, the effects showed an inverse tendency in the presence of TNF-*α* (Figures 3(b) and 3(c)).

**Figure 4:**
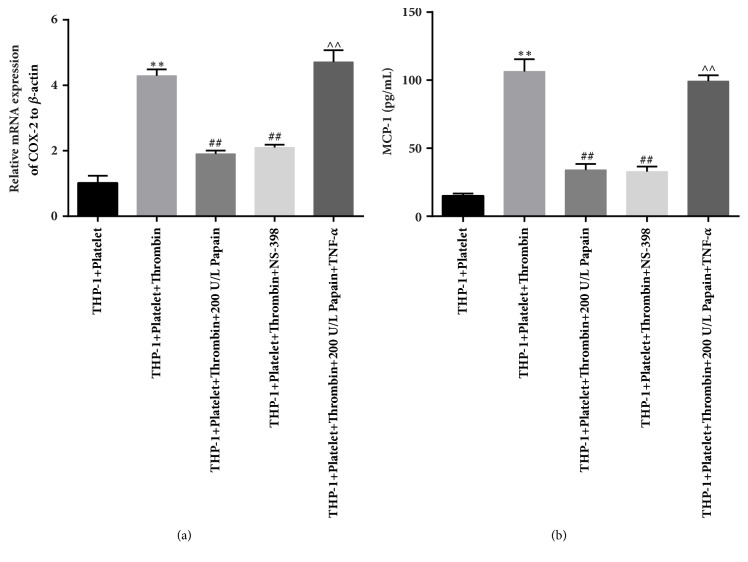
**Impact of TNF-** **α** **on the inhibitory effects of papain on Cox-2 and MCP-1 expression of activated monocytes. **(a) Transcriptional level of Cox-2 was measured by qRT-PCR detection in control, thrombin-induced, and papain, NS-398 as well as TNF-*α*-treated groups. (b) The levels of MCP-1 in the five groups were measured by ELISA kit. K-S test showed that data were in normal distribution in all groups (P>0.05). *∗* indicates thrombin-induced group versus control group. ^#^ indicates papain and thrombin-induced or NS-398-treated group versus only thrombin-induced group. ∧ indicates papain+TNF-*α*-treated group versus papain or NS-398-treated groups. *∗∗*p<0.01, ^##^p<0.01, ∧∧p<0.01.

Eventually, we detected the levels of Cox-2 mRNA and MCP-1 expression under the same conditions to confirm our speculation. As shown in Figure 4, when compared to single thrombin-induced group, both transcriptional level of Cox-2 and the expression of MCP-1 were significantly downregulated by 200U/L of papain or NS-398 (Figures 4(a) and 4(b), the third and fourth columns versus the second one), but TNF-*α* could markedly stimulate the transcriptional level of Cox-2 and the expression of MCP-1 even in the presence of 200 U/L of papain (Figures 4(a) and 4(b), the third and fourth columns versus the fifth one). Therefore, the data suggests that NF-*κ*B signaling-mediated expression of Cox-2 might be involved in the process of MPAs formation-mediated monocytes activation, and papain can inhibit this process.

### 3.4. ERK/p38 and PI3K/Akt Signal Pathway Are Involved in the Papain-Mediated Improvement of Monocytes Activation Initiated by MPAs Formation

**Figure 5:**
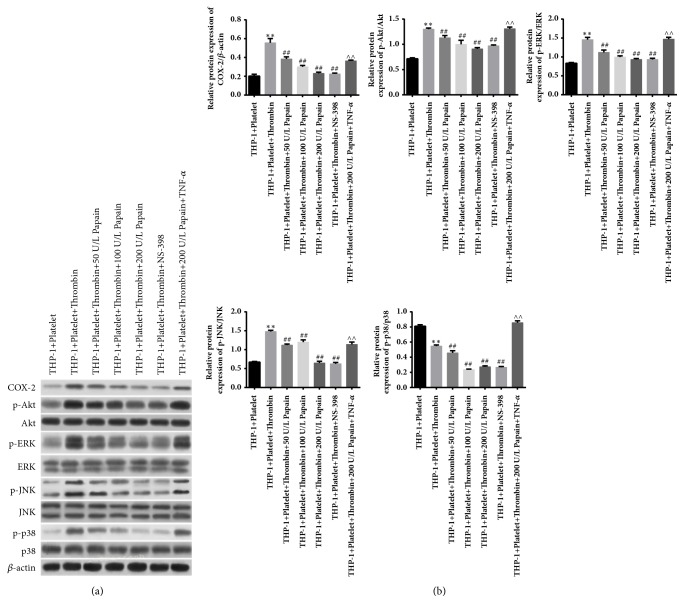
**Investigation of signaling pathway involved in the papain-mediated improvement of monocytes activation initiated by MPAs formation.** (a) Western blotting assay was performed in cocultured system of THP-1 cells and activated platelets by primary antibodies against phosphorylated ERK, p38, JNK, and Akt and the levels of ERK, p38, JNK, Akt, and Cox-2. *β*-actin served as the internal control. (b) Results of quantitative analysis of protein expression of Cox-2/*β*-actin, p-Akt/Akt, p-JNK/JNK, p-ERK/ERK, and p-p38/p38. K-S test showed that data were in normal distribution in all groups (P>0.05). *∗* indicates thrombin-induced group versus control group. ^#^ indicates papain and thrombin-induced or NS-398-treated group versus only thrombin-induced group. ∧ indicates papain+TNF-*α*-treated group versus papain or NS-398-treated groups. *∗∗*p<0.01, ^##^p<0.01, ∧∧p<0.01.

To explore the underlying regulation mechanisms of papain in the process of MPAs formation-initiated activation of monocytes, we performed immunoblotting assay in the cocultured system of THP-1 cells and activated platelets under the treatment with papain, NS-398, and TNF-*α*. It has been verified that MAPKs-NF-*κ*B and PI3K/Akt-NF-*κ*B were implicated in the course of MPAs formation-evoked activation of monocytes. In this study, we discovered that MAPKs signaling cascades such as phosphorylated ERK, p38, and JNK were boosted under the stimulation with thrombin. However, phosphorylation of ERK, p38, and JNK was inhibited upon the treatment of papain and NS-398, and the inhibitory effect of papain was in a concentration-dependent manner (Figure 5(a)). Besides, PI3K/Akt signal pathway was also upregulated in the presence of thrombin, but also repressed by papain and NS-398 (Figure 5(a), the second band). Being consistent with the alteration of MAPKs and PI3K/Akt signaling, the level of Cox-2 expression showed the same tendency with the phosphorylation of signaling molecules (Figure 5(a), the first band). However, administration of TNF-*α* could abate the inhibitory effect of papain on ERK/p38 and PI3K/Akt signal pathway and result in the increase of Cox-2 level (Figures 5(a) and 5(b)). Therefore, ERK/p38-NF-*κ*B and PI3K/Akt-NF-*κ*B signal-mediated expression of Cox-2 might be implicated in the papain-mediated improvement of monocytes activation initiated by MPAs formation.

## 4. Discussion

Atherosclerosis, as a chronic inflammatory disease, is correlated with excessive monocytes activation and a series of inflammatory responses [25]. MPAs formation-mediated monocytes activation acts as the key role in atherosclerosis and thrombosis and is also the facilitators of cardiovascular disease [26, 27], implying that reduction of MPAs formation might contribute to the inhibition of monocytes activation and the prevention and treatment of atherosclerotic cardiovascular disease. In the present study, we discovered that papain prevented MPAs formation and the following activation of monocytes, as well as the release of proinflammatory cytokines from activated monocytes. Therefore, papain could be used as a potential drug for intervention of MPAs formation-mediated inflammatory process or atherosclerotic disease.

Platelet activation is also the key role in the pathogenesis of arterial thrombosis. Plentiful evidence indicated that activated platelets induce the formation of monocyte-platelet complexes in a P-selectin-dependent pathway [28, 29]. Elevation of MPAs levels is the hallmark of platelets activation and contributes to the elevation of activated monocytes, as well as the increase of procoagulant activity and inflammatory response, causing atherogenesis and the development of other inflammatory diseases [12, 30]. Based on the importance of MPAs in inflammatory response, measurement of circulating MPAs has been regarded as a reliable indicator of platelet activation in the context of acute atherothrombosis. Flow cytometry (FCM) is identified as a well-established technique for MPAs detection [31]. Monocytes events are always labeled with the lipopolysaccharide receptor CD14 and activated platelets are gated by the expression of platelet surface marker CD41 [32]. In this study, the thrombin-mediated activation of platelet promoted the increase of MPAs by flow-cytometric quantification analyses using two-color platform (CD14-PE and CD41-APC). However, treatment with papain significantly inhibited the amounts of CD14^+^CD41^+^ cells. Generally, platelet decline is closely associated with inflammatory disease caused by the increased activation of platelet and subsequent sequestration of platelets in cell-cell interactions such as MPAs [33]. Upon platelet activation, the expression of P-selectin is augmented and MPAs are thereby enhanced leading to the reprogrammed monocyte activation through enriching MRP-14 protein [34]. Accompanied by the activation of monocytes, the content of MPAs and the expression of CD11b on monocytes are both promoted in atrial fibrillation (AF) patients with thrombus formation [35, 36]. Here, we also discovered that activated platelet augmented the percentage of CD11b positive cells. Under the condition of papain treatment, the proportion of CD11b positive cells was nearly returned to normal level.

Importantly, activated monocytes initiate the secretion of proinflammatory and atherogenic molecules which facilitate the infiltration of monocytes to the subintima space [37]. Lipopolysaccharide (LPS)-induced monocytes activation such as THP-1 macrophage system promoted expression of proinflammatory genes TNF-alpha and Cox-2 dependent of angiotensin type 1 receptor (AT1R) has essential action in the pathogenesis of atherosclerosis [38]. It was observed that high amounts of Cox-2 in monocytes have been discovered in human atherosclerotic lesions and Cox-2 inhibitor has been actively pursued as an anti-inflammatory reagent in aspirin cardioprotective failure [39, 40]. In the progression of cardiovascular disease, TLR4 (toll-like receptor 4) signaling not only mediates monocyte infiltration, it also promotes the release of inflammatory cytokines including MCP-1 from activated monocytes [41], implying that Cox-2 and MCP-1 expression might serve as the indicators of activated monocytes in CAD events. In our study, under treatment with thrombin, the adhesion rates between THP-1 cells and platelets were elevated along with the increase of Cox-2 and MCP-1 expression levels. The expression of Cox-2 and MCP-1 was markedly inhibited by papain in a concentration-dependent manner. Based on the description above, selective Cox-2 inhibition might contribute to the reduction of inflammation response and probably to the improvement of the inflammation-mediated AS. We further verified the protective role of Cox-2 inhibitor NS-398 as well as papain* in vitro* in the formation of MPAs and monocytes activation. Therefore, these data suggest that papain plays an inhibiting role in MPAs formation and the subsequent monocytes activation which causes the following inflammatory response by regulating Cox-2 expression.

Platelet function disorder is the major risk factor of atherosclerotic cardiovascular diseases. Histones promote platelet aggregation and platelet-dependent thrombin generation by inducing the activation of ERK, p38, Akt, and NF-*κ*B and are important triggers in proinflammatory platelet responses [42]. In the development of hyperuricaemia-related cardiovascular disease, ERK/p38 is activated and PI3K/Akt signal pathway is inhibited in cardiomyocytes and subsequently reduces cardiomyocyte viability [43]. Phloroglucinol administration in platelets is helpful to cardiovascular health by modulating Cox-2 activity through repressing ERK/p38 signaling [44]. In THP-1 cells, norepinephrine (NE) can induce the secretion of matrix metalloproteinase-9 (MMP-9) and augmented the progression of AS through promoting ERK/JNK-c-Fos pathway [45]. Plentiful evidence has also indicated that MAPKs and PI3K-Akt signal pathway can activate the downstream NF-*κ*B and enhance the progression of atherosclerosis [46, 47]. Therefore, ERK-p38-NF-*κ*B and PI3K/Akt-NF-*κ*B signal transduction would be essential in the pathologic process of inflammatory disease such as AS. In the present study, we confirmed that ERK-p38-NF-*κ*B and PI3K/Akt-NF-*κ*B signal pathways were activated and Cox-2 expression increased in the process of MPAs formation-induced activation of monocytes, whereas the signal pathways were significantly suppressed in the presence of papain or Cox-2 inhibitor. Conversely, treatment with TNF-*α*, an activator of NF-*κ*B signal pathway, vividly blocked the inhibitory effect of papain on the MPAs formation, the subsequent monocytes activation, and activation of MAPKs-Akt-NF-*κ*B signal pathway, which indicated that papain potentially attenuated the inflammation response mediated by MPAs formation-initiated monocytes activation by regulating ERK/p38 and PI3K/Akt signal pathway to inhibit Cox-2 expression.

In summary, our study suggests that papain possesses the protective effects on MPAs formation, and the subsequent monocytes activation as well as the following inflammation response by inhibiting Cox-2 expression via regulating ERK/p38 and PI3K/Akt pathway in THP-1 cells. It would be useful to explore the effects of papain on the development and progress of AS through more further studies* in vitro* and* in vivo*.

## Data Availability

The data used to support the findings of this study are available from the corresponding author upon request.
